# Teacher emotion and pedagogical decision-making in ESP teaching in a Chinese University

**DOI:** 10.3389/fpsyg.2022.955474

**Published:** 2022-07-26

**Authors:** Hua Zhao, Danli Li, Yong Zhong

**Affiliations:** ^1^English Department and Research Institute of Foreign Languages, School of Foreign Languages and Literature, Wuhan University, Wuhan, China; ^2^Department of Chinese Studies, School of Humanities and Languages, University of New South Wales, Sydney, NSW, Australia

**Keywords:** teacher emotion, feeling rules, emotion labor, pedagogical decision making, ESP teaching

## Abstract

Teacher emotion has become an important issue in English language teaching as it is a crucial construct in understanding teachers' responses to institutional policies. The study explored teachers' emotion labor and its impact on teachers' pedagogical decision-making in English for Specific Purposes (ESP) teaching in a university of Traditional Chinese Medicine (TCM) in China. Drawing on a poststructural perspective, the study examined data from two rounds of semi-structured interviews, policy documents and teaching artifacts. The analysis of data revealed that the major emotion labor facing the participants revolved around students' disengagement in class. Teachers experienced mixed feelings of anticipation, disappointment, anger, and empathy toward students and distanced themselves from institutional feeling rules enforcing objective assessment of students' performance and punishing students for lack of engagement in class. The study found that teacher emotion labor served as the site for their pedagogical modifications. ESP teachers' beliefs in the importance of attending to students' needs become a powerful discourse in supporting teachers to strategically subvert institutional feeling rules and critically reflect on the dysfunctions of curriculum, orienting teachers' agentic actions in modifying pedagogical practices. We thus underscore this empowering discourse as the bridge to connect teachers' policy negotiation and their actual classroom practices. We also highlight teachers' pedagogical decision-making as a process of the interactions of teacher emotion, teachers' reflexive practices, and power relations. The study ended by suggesting more longitudinal research where teachers' beliefs as previously appropriated discourses could be examined comprehensively as they were both the construct of emotion labor and the potential subverting power in supporting teachers' pedagogical decision-making in policy negotiation.

## Introduction

“We are so in a struggle. The students do not like the courses (medical English courses) and think of them as useless. It is just awkward to give lessons that interest nobody in the class. I do not even want to step into the classroom, yet I know I have to. English for Special Purposes (ESP) is the trend, and the school is determined to implement this reform. It (the reform) is inevitable and is claimed to be beneficial to the development of English teaching in universities in China. But it is just painful for us teachers to face such cold feedback from students in class.” (Teacher A, a college medical English teacher, personal conversation)

Teacher A's remark reveals the emotional struggle that many language teachers face in Chinese universities when the ESP curricula they deliver are not well-received by the students. Despite their passionate implementation of the new ESP curricula, they experienced self-doubt and frustration when students were not interested in the courses they developed for them. Research has documented that teacher emotion not only influences teachers' sense of efficacy (Zheng et al., 2020) but also plays a key role in sustaining their professional development (Day and Leitch, 2001; Golombek and Doran, 2014; Lantolf and Swain, 2019) and identity construction (Lasky, 2005; Wolff and De Costa, 2017; Tao and Gao, 2021). For this reason, it has become an important concern in language teacher education research as “the emotions are educationally central” (Hargreaves, 1998, p. 851).

Teacher emotion becomes an increasingly significant issue for language teacher education researchers as language teachers need to respond to a variety of educational changes in many contexts, such as the shift from English for General Purposes (EGP) to ESP in many Chinese universities (Tao and Gao, 2017; Jiang et al., 2019). The introduction of ESP courses in college English curriculum reform (Xu and Fan, 2017) puts teachers under great emotional stress. It is important to find out how English language teachers deal with emotions and adapt their pedagogical practices.

Previous teacher emotion research has been mainly cognitively oriented, regarding emotions as private psychological states (Han et al., 2021; Li and Liu, 2021; Lei and Xu, 2022) while underestimating their social and political facets (Zembylas, 2003, 2005; De Costa et al., 2020). More recent poststructural approaches view emotion as sociopolitical constructs and situate emotions within the working of power relations (Zembylas, 2011; Benesch, 2012; De Costa et al., 2018). They underscore teacher emotions as agency and subverting strategies rather than private feelings (Benesch, 2017, 2020). On this basis, poststructural approaches yield insights into how teachers' decision-making is entangled with their emotional response to institutional power and suggest a need to “trace the relationship between policy negotiation and actual classroom practice in order to better understand how practitioners' emotional responses to top-down language policies ultimately impact their instruction” (Her and De Costa, 2022, p. 9). To address this end, our study focused on a group of ESP teachers in a Chinese university to explore what emotions are experienced in their adaptation to ESP teaching, how they negotiate their emotions as a response to institutional power, and how this process impacts their pedagogical decision-making in actual classroom practices. We discuss below how teacher emotion is theorized and examined under the poststructural framework.

## Emotions in a poststructural perspective

Feeling rules, along with emotional labor, are a pair of concepts coined by Hochschild (1983) to describe our emotional experiences. By feeling rules, Hochschild means the explicit instructions on what emotions are considered appropriate and are expected and what emotions are considered inappropriate and should be dismissed or repressed at the workplace. They are mirrored illustrations of inequities of power. They help researchers explore how the institutional mandated emotional rules impact workers' internal psychological state as Hochschild assumes an *authentic self* within each individual. That is, workers may find themselves facing the conflict between the mandated and expected feelings and their real felt emotions, and when this happens, they need to make efforts to repress the feelings of their authentic selves and display appropriate emotional expressions. Such display of compulsory feelings, according to Hochschild (1983), is called emotional labor. Hochschild (1983) categorizes workers' efforts in displaying mandated feelings into surface acting and deep acting. While the former represents an attempt at performing certain expected emotions through the change of demeanor without actually feeling them; the latter refers to the deeper management of emotions where mandated feelings not initially experienced are eventually for real.

The poststructural perspective of emotional labor makes a major modification to Hochschild's concept by denying the existence of the *authentic self* and breaking the dichotomy of real and fake self. Tracy and Trethewey (2005) elaborated on their understandings of “self” as constantly constructed and negotiated, and replaced the dichotomy of real and fake self with *preferred self* and *crystallized self* . *Preferred self* refers to the institutionally-formulated identity that employees are supposed to adopt, while *crystallized self* means the multi-dimensional and multi-layered possible selves that are shaped and reconstructed in unpredictable ways. This change of terms opens up space to include how discourses take part in the constant construction and reconstruction of identity. The value of this change also lies in it allowing the process of how individuals “embody, enact, or resist notions of the real and fake” (Tracy and Trethewey, 2005, p. 185) to be examined. Benesch (2017) elaborated on her theorizing of emotion and defined teacher emotion as discursively constructed in the conflicts between institutional feeling rules and teachers' beliefs developed in previous experiences and training. She argued that feeling rules were discourses formed in the workings of power and ideology, and teachers responded to institutional power by adopting and resisting these discourses in their teaching practices. She replaced the word “emotional labor” with “emotion labor” to express a more neutral stance, departing from a cognitive perspective that regards “emotional labor” as a negative term that is opposite to reason. Her theorizing of feeling rules and emotion labor are the conceptual tools used in this study.

## Poststructural approaches to teacher emotions

Poststructural approaches try to address how emotions are shaped by power and how emotion and power shape body and idea, which allows for an examination of how emotions as sociopolitical constructs impact teachers' decision-making in class (Benesch, 2018; De Costa et al., 2020). They focus on what emotions can do rather than what emotions are (Zembylas, 2005) and regard emotion as a lens to examine teachers' response to institutional power for a better understanding of their decision-making process in actual classroom activities.

The poststructural approaches to teacher emotions focus on teacher emotion as empowering tools and subverting strategies that can enhance teacher agency (Benesch, 2018; Miller and Gkonou, 2018; Tao and Gao, 2021) in their responses to institutional power. They utilize multiple conceptual and theoretical tools to argue for this functional feature of emotions and suggest studying emotion in relation to pedagogy (Her and De Costa, 2022) within the complex interactions of multi-level sociopolitical resources (Liu et al., 2022) to reveal the role of emotion and emotional rules in educational contexts (Zembylas, 2005). Claiming there is a *missing conversation* of the discursive, political and cultural facets that *define* the emotional experiences of teachers, Zembylas (2002) elaborated on Raymond Williams's concept of “structures of feeling” and juxtaposed it with notions of discourse analysis and power relations of Foucauldian poststructuralism, arguing that structures of feelings are “particular elements within the more general school culture that subvert the existing social norms and rules” (p. 195). Emotional rules are *historically contingent* and teachers can subvert oppressive emotional rules by their resistance. Benesch (2012) incorporated the feminist and Deleuzian's perspectives to illustrate that teachers' and students' emotions could stick to objects in English language teaching (e.g., quizzes, textbooks, writing assignments, grades). She called for a critical examination of this stickiness so that the emotions and the objects they stick to could “be negotiated toward greater understanding and more productive engagement” (p. 134). Benesch (2017) further argued for the centrality of teacher emotion in English language teaching and called for a critical examination of feeling rules as signposts of unequal power in educational contexts and teacher emotion as empowering tools of teacher agentic actions in response to these unequal power relations.

Based on the above assumption, poststructural approaches have been using teacher emotion as a lens to explore the process of how teachers respond to institutional policy and make decisions in language teaching. Findings suggest that teachers negotiate their emotion labor against institutional feeling rules and draw on language and other affordances to reach their pedagogical goals and meet students' needs (De Costa et al., 2018). This process entails reflexive practices where teachers utilize previously appropriated discourses such as the discourse of “teaching-as-caring” (Miller and Gkonou, 2018) to negotiate institutional feeling rules, critically examine the current policy, and problematize areas that need transformation. Zembylas (2014) noticed this reflexive process and elaborated on the notion of “critical emotional reflexivity” as both a concept and praxis that acknowledges teachers' reflexive process as at bottom emotional and also interrogated the interconnection of emotions, power relations, and reflexive processes. Gkonou and Miller (2021) drew on the notion of emotional capital to address teacher emotion labor as empowering tools in their negotiation of pedagogy. They investigated how teachers' emotional capital can develop through their emotion labor and reflective activities and finally evolve into social and cultural capital that could subvert power in educational reforms and changes. They emphasize teachers' reflection both individually and with others, arguing that it can help them develop emotional capital which can be potential power to challenge long-held values and beliefs. Following them, Her and De Costa (2022) explored how top-down language policy brought related feeling discourses and caused possible sites of emotion labor where the emotional capital of teachers could be developed, reused, and strengthened. Their studies show that teachers' emotion labor and accrued emotional capital are highly contextualized. They also suggest that future research should explore how teachers' actual classroom practices are related to their policy negotiation to understand how teachers' emotional responses to policies influence their instruction (Her and De Costa, 2022). Thus, this study tries to explore ESP teachers' emotion labor in relation to institutional and sociopolitical power relations in a Chinese context and examine how teacher emotions impact teachers' pedagogical decision-making in curriculum reforms. We thus attempted to inform policymakers and teachers about the internal transactions between teacher emotion, policy and classroom activities and further legitimize teacher emotion as capital to subvert dysfunctional policies. The following questions were addressed in the inquiry:

1) What emotion labor did ESP teachers experience when adapting to ESP teaching?
2) How do teachers negotiate their emotion labor in response to the institutional feeling rules?
3) How does this negotiation process of emotion labor influence ESP teachers' pedagogical decision-making?

## Methodology

### Context

The study was situated against the broad context of the nationwide curriculum reforms of College English Teaching in China, entailing a shift from English for general purposes (EGP) to English for specific purposes (ESP). The data were collected from a university of Traditional Chinese Medicine (TCM) located in central China. The university was prestigious in the field of traditional Chinese medicine and covers 15 of the National Administration of Traditional Chinese Medicine key disciplines. Because of a Sino-US cooperative education program specializing in nursing in which students were expected to spend their undergraduate stage in China and proceed with their postgraduate study in America, the university has already been enacting ESP courses for this program for over 10 years. With the trend of integrating ESP into college courses in Chinese universities and a drive to promote the culture of Chinese traditional medicine, the school went a step further and decided to implement ESP courses in all the other disciplines in the year 2020. Thus, students of diverse majors are all required to learn medical English of different duration as part of their college English courses. Such a “one-size-fits-all” policy renders medical English compulsory even for those students with majors that have no relation to medical disciplines. Also, the implementation of medical English has led to a simultaneous reduction in English for general purposes (EGP) courses. When this study began, the new curriculum had been enacted for one semester. Despite institutional determination to embrace ESP and the nearly 10 years of previous ESP teaching experience, the current curriculum reform is not achieving the expected effect, and teachers are facing various challenges, finding themselves trapped in intense emotional struggles.

### Participants

Participants of the interviews were recruited by a snowball sampling method (Cohen et al., 2004). All the participants were introduced by the first participant. To achieve a global understanding of ESP teachers' emotions and considering there were multiple ESP courses for different majors in the target school, the researchers communicated with the first participant to recruit teachers as diversified as possible in terms of the ESP courses they were teaching and the length of their teaching experience in ESP. We recruited nine participants with teaching experiences in different ESP courses (see Table 1).

**Table 1:** Information of participants.

**Pseudonym**	**Educational background**	**Years of ESP teaching**	**ESP courses taught**
T1	- BA in English - MA in English Education	7	Nursing English
T2	- BA in English Education - MA in Linguistics	13	Nursing English
T3	- BA in English - MA in Translation	10	Medical English
T4	- BA in English - MA in English Literature	3	Medical English English for TCM culture
T5	- BA in English - MA in English Education	11	Nursing English
T6	- BA in English Language and Literature - MA in Translation	10	Medical English Translation Medical English Interpreting
T7	- BA in English - MA in Linguistics	2	English for TCM culture
T8	- BA in English Literature - MA in Linguistics	2	Medical English
T9	- BA in English - MA in Applied Linguistics	10	Medical English Nursing English

None of the participants had any medical educational background, and their learning experience had revolved around the English Language. Five teachers had gained extensive experience in ESP teaching and had taught related courses for more than 10 years. Three teachers were relatively new in ESP teaching with about two or three years of experience. Three teachers had taught two types of ESP courses. The above aspects about the participants were considered in the study because capturing prevailing discourses and emotion labor of ESP teachers necessitated the inclusion of a range of responses from teachers with different experiences and identities (Benesch, 2018). Yet, in attempting to identify discourses around ESP teaching, responses are categorized by themes that emerged during the analysis process, and the data excerpts presented are referred to as “R” with consecutive order (such as R1, R2, etc.,), and no specific information of participants' identities are shown with the excerpts.

### Data collection

The data collection contained two rounds of semi-structured interviews (each of about 1 h), policy documents and teaching artifacts. The first-round of interviews tried to identify what types of emotion labor were experienced by participants in ESP teaching. The second round of interviews aimed to explore how teachers' emotion labor impacts the pedagogical decision-making of teachers. As teachers' reports of emotion labor in the first round of interviews mainly revolved around students' disengagement, questions in the second round of interviews were designed around three topics: (1) noticing students' disengagement in class; (2) how students' disengagement makes teachers feel and how it affects their teaching; (3) engaging students in the class. All the interviews were conducted in Chinese. Policy documents concerning ESP courses were collected to further reveal explicit expressions of or implicit allusions to power and feeling rules. Curriculum guidelines, lecture notes, and textbooks were also collected as complementary materials to teachers' descriptions of ESP teaching activities.

### Data analysis

The interviews were transcribed verbatim for repeated reading and coding. A paradigmatic approach was adopted to combine top-down and bottom-up methods to analyze and interpret the interview data (Erickson, 2004), as shown in Table 2. Informed by the poststructural framework, we did not approach each teacher's interviews as an individual case study, rather, we focused on teachers' talking of emotions and the underlying discourses. We borrowed the three categories from Benesch (2020): “(1) emotions; (2) expression of contradictory feelings; and (3) allusions to power, and/or resistance to power” (p. 7) and added one category of “expression of pedagogy” to guide our analysis and interpretation. The first two categories helped us identify participants' emotion labor in ESP teaching and answer the first research question. The third category guided us to capture the process of teachers' negotiation of emotion labor against institutional feeling rules, thus addressing the second research question. The final category was added to generate themes of pedagogical decision-making, which, along with the former three categories, guided our investigation of the final research question: How does this negotiation process of emotion labor influence ESP teachers' pedagogical decision-making? Each transcript was repeatedly read for contents that fit any of the above categories. After the transcripts were organized under the four big categories, we utilized the technique of content analysis to identify sub-themes within each category (Miles and Huberman, 1994). Open coding was first conducted to generate descriptive codes which were then compared in the second coding and further organized into broader themes. Each researcher coded the data separately at first and then exchanged the codes to check for agreement. We worked together to refine the codes and grouped them into meaningful themes.

**Table 2:** A sample of data coding steps.

**Research questions**	**Guiding categories**	**Opening coding**	**Second coding**
1 What emotion labor did ESP teachers experience when adapting to ESP teaching?	1 Emotions →	There is no response from the students, and they all kept silent. This is very uncomfortable. I made an elaborate design of classroom activities to make the lesson more interesting for the students. Yet, no one respects the efforts. Their response made you feel your efforts were meaningless. Such a situation dispels my enthusiasm. (T1, Interview transcript)	Students'disengagement Anger, disappointment, and empathy
	2 Expression of contradictory feelings →	I was really angry because I made such elaborate preparation, yet they did not listen carefully, I felt disappointed. But every class will have such students, students who really do not understand what you are talking about. They just cannot understand it when you speak English. I can understand them. (T5, Interview transcript)	
2 How do teachers negotiate their emotion labor in response to the institutional feeling rules?	3 Allusions to power, and/or resistance to power →	I think one possible reason (for students'disengagement in class) is the overloading class hours regulated by the school for ESP courses. Dedicating such a long time to ESP courses is just to claim and emphasize that we are a joint education program. It is like “to write a new poem, you have to pretend to be sorrow.”(T1, Interview transcript) I would talk with the students (about their disengagement in class) with a caring tone, like an elder and a friend. I would not speak in a harsh way or simply blame them. (T9, Interview transcript)	Reflections Resistance Negotiation
3 How does this negotiation process of emotion labor influence ESP teachers' pedagogical decision-making?	4. Expression of pedagogy →	I understand they care more about College English Test Band 4 (CET-4) and College English Test Band 6 (CET-6), so I would spend some time in class teaching the related content, and when teaching medical English, I would sometimes make a point that: “these few words are also in the scope of CET-4 and CET-6, we must pay attention to” to raise their awareness. (T7, Interview transcript)	Pedagogical decision-making

*The underlined parts refer to codes and themes generated through the top-down method; the highlighted parts refer to codes and themes generated through the bottom-up method*.

In the analysis of the first-round interviews, we aimed to explore the question: (1) what emotion labor did ESP teachers experience when adapting to ESP teaching? Thus, the first two categories borrowed from Benesch (2020): (1) emotions and (2) expression of contradictory feelings guided our coding. We first scanned for instances of different emotional expressions which, in the second coding, were clustered under broader patterns of emotions (such as anger, disappointment, etc.). In this process, we also conducted bottom-up content analysis where one theme stood out by frequently recurring in teachers talking about their emotions—students' disengagement. This salient theme directed our attention to students' disengagement-related emotion labor and feeling rules in the subsequent data collection, analysis, and interpretation.

The analysis of the second-round interviews aimed to answer research questions: (2) How do teachers negotiate their emotion labor in response to the institutional feeling rules? 3) How does this negotiation process of emotion labor influence ESP teachers' pedagogical decision-making? We started with a search for instances of (3) allusions to power, and/or resistance to the power of teachers. A bottom-up content analysis of the identified instances generated descriptive codes of teachers' responses to the feeling rules, which were further grouped into three themes: resistance, negotiation, and reflection. A prominent discourse of “attending to students' needs” emerged across the three patterns, and it was also found to be the major theme under which codes generated in the category of (4) pedagogical decision-making were clustered. Policy documents and curriculum guidelines, lecture notes, and textbooks were read closely to generate themes of explicit or implicit institutional feeling rules.

## Findings

### Emotion labor caused by students' disengagement

A recurring theme in the first-round interviews was that participants' emotion labor revolves around one issue, students' disengagement in class. This theme emerged from the collection of codes concerning students' low participation, misbehavior, absence, and non-completion of homework. The sharp contrast between teachers' ideals for positive feedback from students and disappointment in receiving students' actual responses forms a keynote of the source of respondents' emotion labor.

I think the most stressful emotion comes from students. They give no responses in class, and they do not want to communicate with me. It is awkward, and I always feel like I am talking to myself (R1, 1st interview).

The nine participants perceived students' indifference toward ESP courses and were under great pressure in implementing such an unpopular curriculum in class. They felt “awkward” in receiving such feedback and “always” felt like talking to themselves. Four themes emerged from the respondents' expression of contradictory feelings toward this issue: anticipation, disappointment, anger, and empathy.

I tried to design the lesson in an interesting way and hoped the students would like it. Yet, sometimes, I just felt disappointed. They do not give any responses in the class. One time, I was quite well prepared with various activities to engage them in the class, only to find almost half of the class were absent (R2, 1st interview).

The above response shows that teachers brought to the classroom their anticipation of the teaching effect, yet this anticipation turned into disappointment because students gave no response. They also felt a sense of anger (as shown in R3), out of which they tried to communicate with students about their disappointing performance. In their interactions, they experienced a transformation in their emotion from pure anger and disappointment to a mixed feeling of both anger and empathy.

I would certainly feel a little bit angry. There was one time, during a class break, I tried to ask one student who was playing cell phone in the class, and I took it in a joking tone: “so...it looks like cellphone is indeed more charming than the teacher, right?” Then the boy felt ashamed and said: “No, teacher, the truth is I really cannot understand what you are talking about when you speak in English, I am just so poor in English to understand what is going on in class”. I also got some other feedback like: “I studied Japanese in my senior school instead of English, so I am really not good at English” (R3, 1st interview).

R3's remark indicates respondents' open display of vulnerability (Lasky, 2005) to students in the hope of connecting with them and getting them engaged in future classes. The respondent spoke in a “joking” way to involve the student in a discussion of his misbehavior in class. Yet the student's remark suggests that the poor level of English proficiency displayed by some of them forms a hindrance to their participation in classroom activities. As such, students' inadequate level of English proficiency forms an obstacle in ESP teaching (Jiang et al., 2019). Thus, teachers, though irritated by students' disengagement in class, show a discourse of empathy.

The discourse of empathy can also be found in other responses claiming medical English is hard to learn. R4 comes from a respondent who came across the disappointing performance of students in displaying conversations between doctors and patients in class. She felt quite irritated to find barely any original conversations. But she also felt a sense of empathy as she thought of the mission as “no easy task” because it required both language skills and background knowledge of specific diseases.

It is no easy task, I mean, I asked myself honestly and have to admit that I do not have the confidence to say that I can create an original conversation between a doctor and a patient in such a short time in class...Because it does require not only the ability to organize your language appropriately but also background knowledge concerning the specific disease. Because the doctor is supposed to explain the causes of the disease, for example, why do you have allergies or allergic asthma, and then you need to avoid

those allergies... to tell the truth, I myself would rely on a manuscript for performance, let alone the students (R4, 1st interview).

The transformation of teachers' emotions from pure anger to a mixture of anger and empathy indicates a process where teachers take action to connect with students drawing on their emotion labor. Such connection helps teachers to reflect on the mismatching of students' proficiency and the complexity of ESP courses. Teacher-student interactions offer a discursive space for teachers to conduct reflective thinking. Therefore, teachers should not only try to build emotional bonding with colleagues and administrators (Zembylas, 2011) but also with students.

### Resistance, negotiation, and reflection

Findings in this section reveal that teachers negotiate their emotion labor and reflect on the dysfunctions in the curriculum by drawing on their beliefs in teaching to attend to students' needs. They regard the current ESP curriculum as not answering the demands of students and consciously depart from the feeling rules of being objective in assessing students' performance. They adopt a critical stance to reflect on the causes of students' disengagement at social and institutional levels rather than seeking retribution of students.

#### Resistance to feeling rules and negotiation of emotion labor

There is no explicate description of how students should engage in classroom activities and how teachers should judge students' performance in class in institutional policy. The only document found to be related to students' performance in class is the scoring framework in which daily performances (class attendance, class participation, and homework completion) account for 30% of the total score in one ESP course. Notable about this policy is that there is no detailed reference on how to quantify attendance, class participation, and homework completion.

According to the evaluation system of students, their daily performance in class takes up 30% of their final scores in the courses, and absence and display of disengagement in class would cause score reduction:

The course evaluation shall include formative evaluation and summary evaluation with a total of 100 points. The midterm examination accounts for 10%; the formative evaluation accounts for 30%, including class attendance, participation, and assignments; the final examination accounts for 60% in the form of a closed-book exam (excerpts from syllabus).

The scoring system puts teachers in the chair of class manager (Benesch, 2017) and assessor with a compendious description that outlines two dimensions of students' performance in which formative evaluation accounts for 30% of the final score. Such a system requires that teachers objectively observe students' daily performance concerning their class attendance, class participation, and homework completion, and make honest assessments. Thus, teachers are to strictly stick to this framework in synthesizing the scores of formative evaluations that can be seen as praise for those with a good performance in their daily learning and yet a price paid by those who behave in a disappointing manner. As such, the themes of objectivity and retribution emerge as the main feeling rules in teachers' encounters with students' disengagement from the courses.

R5 further indicates the feeling rules of retribution. There is a consensus that frequent absence (over three times) and severe misbehavior in classrooms should cause disqualification for the final exam and invalidation of credit.

Strictly speaking, they should be deprived of the right to participate in the final examination and shall re-study the course for credit if they are absent from the class over three times or perform other severe misbehavior in classrooms (R5, 1st interview).

Thus, teachers are obliged to report severe misbehavior to the school when necessary. Yet, interestingly, there are no detailed illustrations found in the institution of what should be taken as “severe misbehavior” here, and the word “strictly” indicates teachers' perception of the implicit rules as having room for accommodation, which serves as one of the potential sources of emotion labor of teachers, as discussed in the next section.

However, responses suggest teachers make flexible decisions in assessing students' efforts in learning instead of intentionally seeking retribution of students. They tend not to obey the scoring frame dogmatically. As in R6, the respondent expressed that it is “impossible” for her to fail a student who has passed the final examination by a low score on daily performance, which is a clear illustration of her distancing from the institutional feeling rules of objectivity in assessing students' daily performance.

It really depends, I will first check my record for his/her (student) daily performance and relate it to the score of his/her final examination. If he/she scores relatively high in the final examination, it means he/she did make an effort in learning. In that case, I would not give him/her a score that is too low to gain credit for the course, even if the daily performance is indeed disappointing. I mean, at least, I will make sure he/she passes the examination. It is impossible for

performance. I will refer to and respect his/her score in the final examination (R6, 2nd interview).

The above response shows an evident violation of institutional feeling rules of objectivity and retribution. Despite the unpleasant feeling of coming across students' disengagement in class, respondents chose to “respect” students' scores on the final examination. The reason for such departure from the feeling rules may lie in the ambiguity of the policy (with no detailed description on how to quantify students' performance in class) which had led to teachers' “lack of conviction about the gravity of the incident” (Benesch, 2020, p. 7). Notable in the remark is that teachers jump out of their mandated role as enforcers and take up a soft stance for a comprehensive evaluation of students' learning reality, revealing that teacher emotion labor is the site of identity transformation (Zembylas, 2003) and also indicating that teachers' identity commitment lies behind their affective reactions to their work and the settings (Nias, 1996).

#### Reflections and the discourse of attending to students' needs

The following responses indicate that teachers reflect on external institutional and sociopolitical contributors (employment market, and English grade examination) to students' disengagement in ESP courses, drawing upon the discourse of teaching to attend to students' needs. This does not mean that students' misbehavior in class is neglected. Instead, it is treated with equanimity and rationality (Benesch, 2018). R7 comes from respondents who teach students from the Sino-US cooperative education program of the nursing specialty, suggesting graduates of nursing in their school enjoy a priority in the local job market and barely choose to continue further education abroad. English is not needed for future work, and for this reason, students were not motivated to learn it.

One of our teachers tries to confabulate with students (about the issue of students' disinterest in English), and their remarks are quite interesting. Students think of these courses as useless, especially the listening and speaking parts. Students express directly that it is impossible for them to have a chance to receive foreign patients and speak English with them. I mean they are well-received by the job market, so even if we call their program Sino-US cooperative education, they barely choose to pursue further education overseas, they just graduate from our school and find a job at the local grade A hospitals. They have such a good prospect of finding a job that they do not need English (R7, 2nd interview).

In R7, the respondent thinks the reason given by students concerning their disinterest in medical English is “interesting,” which indicates a clear departure from institutional feeling rules of retribution. Instead of a punitive stance, teachers “confabulate with” students in search of the possible crux of the issue. The word “confabulate” suggests a caring attitude of teachers against the institutional discourse of retribution.

R8 indicates another mismatch between students' needs in passing CET-4 and CET-6 and the current ESP curriculum. For students of majors other than English, those two are significant tests to verify their English proficiency and are often referenced by employers in the job-hunting process. Yet, medical English vocabulary is considered “obscure” and not directly helpful in the above examinations.

They may think it is unnecessary to learn the course. I mean, if it is general English courses, we have words that are needed for CET-4 and CET-6. Yet, for medical English, most of the words are obscure and barely used in daily life. Students see no use in learning them. After all, the words will not be helpful in CET-4 and CET-6, so they are reluctant to invest too much energy in the course (R8, 2nd interview).

Both R7 and R8 associate students' disengagement with possible deficiencies in ESP teaching policies and curriculum. Teachers' belief in attending to students' needs becomes an empowering discourse for teachers to critically examine whether the course prepares students for language use in real-world conditions and whether they need English for future work. They understood why students became disengaged in the learning process as they recognized that the ESP courses do not address students' needs. In the above process, this discourse had been reinforced and would further impact teachers' pedagogical decision-making in ESP teaching, as discussed in the next section.

### Pedagogical decision-making

Teachers' negotiation of emotion labor has reinforced the discourse of “attending to students' needs” which further orients their pedagogical decision-making in class. The following responses indicate that seeking pedagogical solutions and not retributions of students was the preferred stance taken by respondents. Teachers reached out to meet students' practical needs drawing on different pedagogical approaches.

Actually, now we are trying to make our course an “excellent course,” so we are working on online micro-courses from which students can preview the content of each lesson before the actual class to get a primary understanding of what will be taught in class. Following this preview, they

come to the class with a basic impression of the content for further detailed learning (R9, 2nd interview).

As mentioned in the previous section, respondents showed empathy toward students in learning medical English which is harder than general English and contains many obscure words, especially toward those students with poor competence in English. So, they tried to make the learning process easier. R9 indicates teachers were trying to establish a system where the content of lessons is gradually released. They were trying to put micro-courses online to offer students a chance to preview the content, in the hope of helping them develop a deeper understanding in class.

R10 describes a strategy of relating medical English to daily English, using the meaning of daily English to promote students' understanding of word usage in medical English.

Like the word “terminal,” it means “the final phase of a disease,” right? It is also used for other meanings in our daily life. In the class, I will first introduce its basic meaning as in CET-4 and CET-6, and then explain its meaning in medical English (R10, 2nd interview).

In addition, R11 reports an even deeper reflection of teachers on pedagogy at the institutional level. The respondent believes ESP courses should be conducted under graded teaching where students' diversity in English proficiency can be considered. Students should be given an English test right after their entrance into universities and the option of choosing English lessons that are suitable for their levels. This is an overt resistance to the current institutional policy which signals respondents' in-depth reflection and engagement in the negotiation of ESP teaching policies and practices. Also, it verifies that emotions are interwoven with power relations and reflexivity (Zembylas, 2014).

I think it will be better for us to conduct graded teaching...for the students with relatively high proficiency, you can speed up your pace in class and choose more complex content.... many other schools are doing graded teaching. Students can choose teachers and the time of class in the school system. In that way, students are not constrained by the class-cohorts tried to their major. Every student will be given an English test at the beginning of their future college English lessons, and they will be assigned to different levels of English proficiency, which can be a reference for them in choosing suitable lessons. And the lessons available should be diversified both in content and class hour (R11, 2nd interview).

These responses indicate that teachers have taken action in trying pedagogical solutions in facing students' unsatisfactory performance in class. They tried to attend to students' needs in place of accusing and punishing them, which reveals that the reinforced discourse of teaching to attend to students' needs had developed into an emotional capital (Gkonou and Miller, 2021) to orient teachers' pedagogical decision-making in actual teaching activities. Issues like students' disengagement should be considered critically, and teachers should take into consideration students' uneven level of English proficiency and utilize different ways to engage students in class (e.g., relating medical English vocabulary to daily-use English to facilitate students' understanding).

## Discussion

The study draws on a poststructural lens to explore teachers' emotion labor and their pedagogical decision-making in ESP teaching practices. The analysis of ESP teachers' interview data revealed that students' disengagement serves as the major source of teachers' emotion labor. A sharp contrast between anticipation before the class and disappointment in perceiving students' disengagement was reported. Institutional feeling rules toward students' disengagement are found to be objectivity and retribution. Yet respondents tend to resist such feeling rules and experience a mixed feeling of anger and empathy, with no punitive intentions. They reach out to students' needs and try to modify their pedagogy in class instead of seeking retribution of students.

The findings of the study suggest teachers' belief in teaching to attend to students' needs bridges teachers' policy negotiation and their pedagogical decision-making in actual classroom activities. It works not only as a construct of teacher emotion labor but also as an empowering discourse in supporting teachers to negotiate their emotion labor against institutional feeling rules, critically reflect on the discrepancies between the current curriculum and students' real needs in class, and make decisions for their pedagogical practices. The findings corroborate that emotion labor plays “an important role in shaping teachers' pedagogical practices in the classroom” (De Costa et al., 2020, p. 211) and deepen our understanding of how emotion labor “can accrue as emotional capital” (Gkonou and Miller, 2021, p. 150) for teachers to negotiate feeling rules and pedagogy. Yet the accrued emotional capital is highly contextualized (Her and De Costa, 2022) and interwoven with reflexive practices and power relations (Zembylas, 2014). Teachers' accruing of emotional capital and their policy negotiation are an iterative process in which the discourse that empowers teachers to resist feeling rules and conduct reflexive practices can be reinforced and developed in turn by these discursive practices to further facilitate teachers' policy negotiation and drive teachers' agentic action on modifying their pedagogy. Therefore, (1) teachers should be informed of the importance of “critical emotional reflexivity” (Zembylas, 2014) and researchers should acknowledge teachers' policy negotiation and pedagogical decision-making as a process of the interplay among teacher emotion, reflexive practices, and power relations; (2) teachers' previously appropriated discourses should be examined in our inquiry on teachers' agentic engagement in pedagogical renewals, and teacher training programs should pay attention to the ideology and discourse they convey to teachers as they play an important role in teachers' coping with emotion labor and adopting pedagogical strategies in the future; (3) social ecologies in which teachers' work is embedded should be examined in critically-inflected teacher emotions research (De Costa et al., 2018) for a contextualized understanding of teachers' emotion labor and their pedagogy negotiation;

The study also suggests teachers-students emotional connections serve as discursive practices where teachers' belief in teaching to attend to students' needs is reinforced and developed into alternative discourses against institutional feeling rules and further facilitate their problematizing of the current pedagogy and policy. This discovery corroborates that teacher's relationship with students is “grounded in empathy and pedagogical responsibility” (Benesch, 2018, P. 9). Teachers' emotional resonance with students provides ways for them to “know their teaching, themselves and others” (Zembylas, 2003, p. 233). Thus, we should highlight the importance of teachers establishing “emotional affinities” (Zembylas, 2003) with students. That is, teachers should not only draw on emotion labor to connect and bond with colleagues but also should be closely connected to students to extend the scope of “collective agency” (Tao and Gao, 2021) in institutions.

The above implications are rather significant in our understanding of teachers' work under current ongoing curriculum reform from EGP to ESP teaching as the policy of ESP teaching is in turbulence with constant revision. Concerns have been increasing about how teachers respond to these changes, cope with emotional struggles and modify their pedagogical practices in such context. Seeing teacher emotions from a poststructural perspective can provide us an insight into ESP teachers' professional development and help us understand ESP pedagogy.

## Conclusion

The study tries to answer the calls for exploring teacher emotion labor from a poststructural perspective against different educational contexts and contributes to our understanding of how teachers' practices in classrooms are impacted by their emotional responses to institutional feeling discourses. It added to our knowledge about the relationship between teacher emotion, policy negotiation, and pedagogical practices and highlighted the importance of reflexivity in the development of teachers' emotional capital in facing institutional power. The study also underscored the role teachers' previously appropriated discourses play in their negotiation of emotion labor against institutional feeling rules.

However, what the study did not accomplish is a longitudinal observation and examination of teachers' previous life experiences and their relationship with their emotions, which needed further investigation because teachers' previous experiences play a key role in forming their beliefs which are part of the constructs of emotion labor (Benesch, 2017) and teaching activity is inevitably connected with teachers' personal lives and teacher identity commitments that emerge from teachers' life histories (Tao and Gao, 2017).

## Data availability statement

The raw data supporting the conclusions of this article will be made available by the authors, without undue reservation.

## Ethics statement

The studies involving human participants were reviewed and approved by Ethics Committee, School of Foreign Languages and Literature, Wuhan University, China. The patients/participants provided their written informed consent to participate in this study.

## Author contributions

HZ: conceptualization, methodology, investigation, data analysis, and draft. DL: conceptualization, data analysis, revision, supervision, and funding. YZ: revision and editing. All authors contributed to the article and agreed to the submitted version.

## Funding

This work was supported by the Humanities and Social Science Research Foundation of Chinese Ministry of Education (Grant Number: 19YJA740025) and the Fundamental Research Funds for the Central Universities in China (Wuhan University, Grant Number: 2020AI006, 1103-413000094).

## Conflict of interest

The authors declare that the research was conducted in the absence of any commercial or financial relationships that could be construed as a potential conflict of interest.

## Publisher's note

All claims expressed in this article are solely those of the authors and do not necessarily represent those of their affiliated organizations, or those of the publisher, the editors and the reviewers. Any product that may be evaluated in this article, or claim that may be made by its manufacturer, is not guaranteed or endorsed by the publisher.
